# Excessive fear of clusters of holes, its interaction with stressful life events and the association with anxiety and depressive symptoms: large epidemiological study of young people in Hong Kong

**DOI:** 10.1192/bjo.2023.540

**Published:** 2023-08-14

**Authors:** Stephanie M. Y. Wong, Eric Y. H. Tang, Christy L. M. Hui, Y. N. Suen, Sherry K. W. Chan, Edwin H. M. Lee, K. T. Chan, Michael T. H. Wong, Arnold J. Wilkins, Eric Y. H. Chen

**Affiliations:** Department of Psychiatry, School of Clinical Medicine, LKS Faculty of Medicine, The University of Hong Kong, Hong Kong; Department of Psychiatry, School of Clinical Medicine, LKS Faculty of Medicine, The University of Hong Kong, Hong Kong; and The State Key Laboratory of Brain and Cognitive Sciences, The University of Hong Kong, Hong Kong; Department of Psychology, University of Essex, Essex, UK

**Keywords:** Trypophobia, epidemiology, anxiety symptoms, depressive symptoms, stressful life event

## Abstract

**Background:**

Excessive and persistent fear of clusters of holes, also known as trypophobia, has been suggested to reflect cortical hyperexcitability and may be associated with mental health risks. No study, however, has yet examined these associations in representative epidemiological samples.

**Aims:**

To examine the prevalence of trypophobia in a population-representative youth sample, its association with mental health and functioning, and its interaction with external stress.

**Method:**

A total of 2065 young people were consecutively recruited from a household-based epidemiological youth mental health study in Hong Kong. Trypophobia, symptoms of anxiety, depression and stress, and exposure to personal stressors were assessed. Logistic regression was used to assess the relationships between trypophobia and mental health. Potential additive and interaction effects of trypophobia and high stress exposure on mental health were also tested.

**Results:**

The prevalence of trypophobia was 17.6%. Trypophobia was significantly associated with severe symptoms of anxiety (odds ratio (OR) = 1.83, 95% CI = 1.32–2.53), depression (OR = 1.78, 95% CI = 1.24–2.56) and stress (OR = 1.68, 95% CI = 1.11–2.53), even when accounting for sociodemographic factors, personal and family psychiatric history, resilience and stress exposure. Dose–response relationships were observed, and trypophobia significantly potentiated the effects of stress exposure on symptom outcomes, particularly for depressive symptoms. Those with trypophobia also showed significantly poorer functioning across domains and poorer health-related quality of life.

**Conclusions:**

Screening for trypophobia in young people may facilitate early risk detection and intervention, particularly among those with recent stress exposure. Nevertheless, the generally small effect sizes suggest that other factors have more prominent roles in determining recent mental health outcomes in population-based samples; these should be explored in future work.

Trypophobia has been described as an excessive and persistent fear of clusters of holes.^1^ Spectral features of visual stimuli characterised by high-contrast energy at midrange spatial frequencies have been considered to underlie the condition.^1,2^ A recent study has suggested that despite the influences of amplitude spectrum (i.e. deviation from the 1/f spectrum as in natural images), the phase spectrum of trypophobic stimuli (i.e. circular clusters of small objects and holes) plays a more prominent role in the experience of trypophobic discomfort.^3^

Despite having been documented in earlier case reports^4^ and on online forums,^5^ the phenomenon has only received attention in the scientific literature and appeared in online search engine results in 2015^5^ when the Trypophobia Questionnaire^4^ was developed. Notably, although trypophobia is associated with ‘phobias’, few studies have examined the relationship between trypophobia and mental health.^5–7^ A number of recent studies have found physiological markers, including increased heart rate variability, cortical haemodynamic responses and pupillary constriction, to be associated with trypophobia.^8,9^ Whether trypophobia could serve as a brief transdiagnostic marker of mental health risks with functioning implications remains to be determined.

Trypophobia symptoms can generally be categorised into three domains, namely skin-related (e.g. feelings of ‘itchiness’, ‘skin crawl’), cognitive (e.g. aversion, disgust or repulsion’, ‘uneasy’) and physiological (e.g. ‘sick or nauseous’, ‘trouble breathing’) and are most commonly assessed using the Trypophobia Questionnaire.^5^ According to Cole and Wilkins (2013),^1^ these symptoms may reflect non-conscious responses towards stimuli that possess low-level and easily computed spectral compositions, similar to characteristics of potentially ‘dangerous’ organisms such as snakes. Other studies have also observed associations between trypophobia symptoms and cluster-like circular patterns characteristic of infectious diseases, such as skin lesions and infection-related scars.^10^ The associations with visual stress as reported in prior work (as a reflection of cortical hyperexcitation) have led to the hypothesis that symptoms of trypophobia may help reduce load on the visual cortex by encouraging avoidance.^8,10,11^ Although trypophobia symptoms may initially be considered to be automatic adaptive responses to danger in the environment, modern examples of trypophobic stimuli are not necessarily life-threatening (e.g. lotus seed pods and honeycombs).^8^ The condition has therefore also been conceptualised as a form of ‘overgeneralised’ avoidance response.^10^ A recent study using an adapted version of the Trypophobia Questionnaire has extended existing work on adult and youth samples to show that trypophobic discomfort is present in children as young as 4 years of age.^12^

Compared with its relationships with physiological conditions, the role of trypophobia in mental health has received little attention in the literature. Existing findings on the relationships remain inconsistent. Two studies have reported non-significant associations between trypophobia and generalised anxiety symptoms,^12,13^ although both were conducted in small samples of undergraduate students (*n* = 37 and *n* = 147, respectively). The original study by Le et al (2015)^4^ reported a weak relationship between Trypophobia Questionnaire scores and trait anxiety among participants from a web-based support group (*n* = 155) but not in a separate university student sample (*n* = 117).^4^ Potential differences in trypophobia symptoms between the samples and exclusion of participants with a higher trypophobia score (Trypophobia Questionnaire score >34) from the university sample may have contributed to these differences.

By contrast, another study found trypophobia to be associated with greater distress and comorbid generalised anxiety disorder and major depressive disorder in a web-based sample.^7^ Of note, some researchers have distinguished between states and traits of anxiety and found that trait anxiety was in fact not significantly associated with trypophobia symptoms,^13^ suggesting that trypophobia symptoms may be reflective of enhanced neurobiological sensitivity to one's external environment rather than psychopathology. Indeed, trypophobia has further been found to be a significant mediator between social anxiety symptoms and discomfort induced by ‘clusters of eyes’ in human faces.^6^

These studies, however, have been limited to diverse online and student samples.^5,10^ The findings thus remain inconclusive. A more comprehensive study of the associations between trypophobia and mental health risks, as well as its possible interactions with environmental factors, is necessary to understand this phenomenon in greater depth and to determine its potential clinical implications for early detection and intervention in the youth population.

Young people are most vulnerable to the onset of mental disorders^14,15^ but are also the least likely to seek help.^16^ The identification of non-stigmatising risk markers in youth populations can therefore be critical for facilitating early engagement work. Trypophobia represents a potentially suitable candidate: not only is it commonly discussed on online forums,^5^ but it is also not officially considered to be a form of specific phobia according to existing diagnostic systems.^17^

To the best of our knowledge, no study has yet examined the phenomenon of trypophobia in a large population-representative sample and how it may interact with external stressors to affect mental health. We therefore conducted a study first to establish the prevalence of trypophobia in the youth population of Hong Kong. We also further aimed to examine: (a) whether trypophobia was associated with severe symptoms of not only anxiety but also of depression and stress, even when accounting for a wide range of other intrinsic and extrinsic risk factors; (b) whether trypophobia would not only add to but also potentiate the effect of external stressors on mental health; and (iii) whether trypophobia would also be significantly associated with poorer functioning and quality of life.

## Method

### Participants

A total of 2065 young people were consecutively recruited between July 2020 and June 2022 as part of the ongoing Hong Kong Youth Epidemiology Study of Mental Health (HK-YES). The HK-YES is the first large-scale household-based epidemiological study of youth mental health in Hong Kong and adopts a stratified multistage cluster sampling design to improve sample representativeness. Invitation letters were mailed to addresses provided by the local government, which were randomised and stratified by geographic location and housing quarter. All young people between 15 and 24 years old at the time of recruitment were invited. All assessments were conducted by trained research staff through face-to-face interviews, or through online video conferencing following the same procedures during COVID-19. Details of the HK-YES have been reported elsewhere.^18–23^

Written informed consent was obtained from all participants, with consent obtained from parents or legal guardians for those below the age of 18. The authors assert that all procedures contributing to this work comply with the ethical standards of the relevant national and institutional committees on human experimentation and with the Helsinki Declaration of 1975, as revised in 2008. All procedures involving human subjects and/or patients were approved by the Institutional Review Board of the University of Hong Kong/Hospital Authority Hong Kong West Cluster (reference number: UW 19-017).

### Measures

#### Trypophobia

Symptoms of trypophobia were assessed using the 17-item Trypophobia Questionnaire,^5^ which has been shown to be a valid measure of trypophobia and has been applied in both experimental and observational studies.^2,6,13^ Following the standard procedures of the Trypophobia Questionnaire, participants were first presented with two trypophobic stimuli (images of a lotus seed head and a honeycomb) and were asked to rate whether symptoms in the cognitive, skin-related and physiological domains were experienced (e.g. ‘aversion, disgust or repulsion’, ‘anxious, full of dread or fearful’, ‘skin crawl’, ‘sick or nauseous’ and ‘trouble breathing’). Items were rated on a five-point Likert scale (from ‘not at all’ to ‘extremely’) and summed to generate a composite score. As altering the threshold resulted in no clear difference in the association between trypophobia and mental health outcomes, the conventional cut-off (score above 31) was used to denote the presence of trypophobia.^5^ The internal consistency of the Trypophobia Questionnaire was excellent (0.94) in this study.

#### Anxiety, depressive and stress symptoms

Symptoms of anxiety, depression and stress (in the past week) were assessed using the 21-item Depression, Stress and Anxiety Scales (DASS-21), which comprises three seven-item subscales for the three symptom dimensions, respectively (DASS-A, DASS-D and DASS-S).^24^ Each item was assessed using a four-point Likert scale (from ‘did not apply to me at all’ to ‘applied to me very much/most of the time’). The composite scores for anxiety, depressive and stress symptoms, respectively, were determined by multiplying the sum of items of each subscale by a value of 2. As in prior work, the conventional cut-offs of 15, 21 and 26 were used to reflect severe depressive, anxiety and stress symptoms, respectively.^24^ The scale and its subscales have been validated and used in Chinese community and youth samples.^22,25–27^ DASS-A, DASS-D and DASS-S also showed good internal consistency in this study (α = 0.82–0.87).

#### Resilience and personal stressful life events

Resilience was assessed using the ten-item Connor–Davidson Resilience Scale (CD-RISC-10).^28,29^ Items were rated on a five-point Likert scale (from ‘not true at all’ to ‘true nearly all the time’). Its Chinese version has been validated in local youth samples^30^ and has also been used in prior work on the Hong Kong youth population.^21^ The internal consistency of the CD-RISC-10 was also excellent in this study (α = 0.90).

Personal stressful life events (SLEs) were assessed using a modified version of the List of Threatening Experiences,^31^ which captures 12 categories of personal stressful events during the past year in a checklist format (yes/no ratings), such as a major financial crisis, loss of a steady relationship, and serious illness or injury to self or a close relative. The options ‘being sacked from job’ and ‘unemployment’ were each given the alternatives of ‘expelled from school’ and ‘dropped out of school’, respectively, to adapt to the current youth sample. An ‘others’ option was also provided, yielding a maximum of 13 SLEs in this study. High stress exposure was defined in this study as the experience of two or more SLEs.

#### Functional impairment, global distress and health-related quality of life

Functional impairment was determined using three items assessing the number of days during the past 30 days that the presence of psychiatric symptoms has affected (a) school or work, (b) social life and (c) household responsibilities. Global distress was assessed using the six-item Kessler Psychological Distress Scale,^32^ where items were rated on a five-point Likert scale (from ‘none of the time’ to ‘all of the time’). Health-related quality of life was assessed using the 12-item Short-Form Health Survey,^33,34^ which consists of two component scales (PCS-12 and MCS-12) that can be used to generate summary scores for physical and mental health-related quality of life (α = 0.74 and α = 0.85, respectively).

#### Adverse childhood experiences

As in previous studies,^35–37^ five domains of family-related adverse childhood experiences prior to the age of 17 years were assessed: parental psychopathology (e.g. had a serious mental health problem, alcohol or drug problem, or attempted or died by suicide), neglect (e.g. inadequate supervision by caregiver or having to do dangerous and age-inappropriate tasks), emotional abuse (e.g. been insulted, told hurtful things or emotionally abused by caregiver), physical abuse (e.g. being pushed, hit or bruised by caregiver) and sexual abuse (e.g. experienced sexual assault or unwanted sexual contact or abuse by caregiver). Each item was rated on a five-point Likert scale (from ‘never’ to ‘very often’). A rating of 3 or above (‘often’ to ‘very often’) was used to denote the presence of adverse childhood experiences within each of the five domains, which were then summed to generate a composite score (ranging from 0 to 5).

### Statistical analysis

The prevalence of trypophobia in the Hong Kong youth population was first established with post-stratification weights applied according to age and sex data from the 2019 local census. In the first set of analyses, we examined the associations between trypophobia and categorical symptom outcomes (severe depressive, anxiety and stress symptoms) with the aim of determining the potential utility of trypophobia in detecting high-risk youths. First, chi-squared tests were conducted to examine these univariate associations. The univariate associations between trypophobia and personal background factors (age, sex, personal and family psychiatric history, childhood adversity), resilience and stress exposure (SLEs), as well as functioning, distress and quality of life, were also tested using independent-samples *t*-tests (continuous variables) or chi-squared tests (categorical variables). Next, separate multivariable logistic regression models were used to determine the contribution of trypophobia to each of the three severe symptom outcomes, accounting for background factors, resilience and SLEs. Effect sizes from all logistic regression analyses are presented in the form of adjusted odds ratios (ORs) with 95% confidence intervals, with the pseudo-*R*^2^ (the squared correlation between predicted and observed values^38,39^) also computed.

As well as using categorically defined severe symptoms as an outcome for detecting high-risk youths, we conducted an additional set of analyses to examine the potential influences of trypophobia on the severity of the three symptom outcomes. To further consider the potential interaction between trypophobia and stress exposure and any effects on symptoms, we conducted two-way analysis of variance (ANOVA) for the presence of each of the three mental health conditions, adjusting for the same set of covariates. Significant main effects would reflect a dose–response relationship, whereas significant interactions would suggest a potentiating effect. Differences in trypophobia symptoms and other sample characteristics between those with and without severe anxiety, depressive and stress symptoms, respectively, are presented in Supplementary Table 1 available at https://doi.org/10.1192/bjo.2023.540. In all analyses, *P* < 0.05 was considered to indicate statistical significance. All analyses were performed using SPSS version 26.0.

## Results

### Prevalence of trypophobia in the Hong Kong youth population

The weighted prevalence of trypophobia was 17.6% in the youth population of Hong Kong. Sample characteristics and differences between those with and without trypophobia are presented in Table 1. Those with trypophobia were more likely to be older (mean = 20.08, s.d. = 2.65 *v.* mean = 19.69, s.d. = 2.83) and female (68.1%); both *P* < 0.05. No significant differences in background factors, including personal and family psychiatric history and childhood adversity, as well as exposure to recent personal SLEs, were found between those with and without trypophobia (*P* > 0.05) (Table 1).

**Table 1:** Sample characteristics and differences between participants with and without trypophobia in the epidemiological youth sample (*n* = 2065)

	Whole sample (*n* = 2065)	Without trypophobia (TQ < 31) (*n* = 1683)	With trypophobia (TQ ≥ 32) (*n* = 382)	*t*	*P*	Effect size
Background factors						
Age	19.76 (2.80)	**19.69** (**2.83)**	**20.08** (**2.65)**	**−2.60**	**0**.**010**	**−0**.**14**
Sex, *n* (%)				–	**<0**.**001**	**0**.**10**
Male	865 (41.9)	**743** (**44.1)**	**122** (**31.9)**			
Female	1200 (58.1)	**940** (**55.9)**	**260** (**68.1)**			
Has personal psychiatric history, *n* (%)	175 (8.5)	142 (8.4)	33 (8.6)	–	0.90	0.001
Has family psychiatric history, *n* (%)	270 (13.1)	215 (12.8)	55 (14.4)	–	0.40	0.02
Has childhood adversity, *n* (%)	711 (34.4)	564 (33.5)	147 (38.5)	–	0.065	0.04
Intrinsic and extrinsic factors						
Resilience (CD-RISC-10)	23.90 (6.24)	**24.08** (**6.17)**	**23.14** (**6.50)**	**2.66**	**0**.**008**	**0**.**15**
≥2 SLEs, *n* (%)	409 (19.8)	323 (19.2)	86 (22.5)	–	0.14	0.03
Severe psychiatric symptoms, *n* (%)						
Anxiety symptoms (DASS-A ≥15)	246 (11.9)	**173** (**10.3)**	**73** (**19.1)**	–	**<0**.**001**	**0**.**10**
Depressive symptoms (DASS-D ≥21)	199 (9.6)	**141** (**8.4)**	**58** (**15.2)**	–	**<0**.**001**	**0**.**09**
Stress symptoms (DASS-S ≥26)	136 (6.6)	**96** (**5.7)**	**40** (**10.5)**	–	**<0**.**001**	**0**.**07**
Functioning, distress, and quality of life						
Functional impairment (past 30 days)						
Work/school	1.21 (1.84)	**1.15** (**1.80)**	**1.44** (**2.00)**	−**2.58**	**0**.**010**	−**0**.**15**
Social life	0.86 (1.61)	**0.81** (**1.57)**	**1.06** (**1.77)**	−**2.49**	**0**.**013**	−**0**.**15**
Household responsibilities	0.79 (1.60)	**0.74** (**1.56)**	**0.99** (**1.76)**	−**2.58**	**0**.**010**	−**0**.**15**
Global distress (K6)	7.45 (5.18)	**7.16** (**5.10)**	**8.73** (**5.33)**	−**5.38**	**<0**.**001**	−**0**.**30**
Physical health-related quality of life (PCS-12)	52.33 (7.15)	**52.49** (**7.00)**	**51.59** (**7.77)**	**2.09**	**0**.**037**	**0**.**12**
Mental health-related quality of life (MCS-12)	41.15 (11.69)	**41.69** (**11.71)**	**38.78** (**11.33)**	**4.40**	**<0**.**001**	**0**.**25**

Descriptive statistics of the sample are presented in the form of mean (s.d.) unless otherwise stated. All values are unweighted. Values significant at the *P* < 0.05 level are shown in bold. Effect sizes for independent samples *t*-test and χ² test were Cohen's *d* and Cramér's *V*, respectively.

CD-RISC-10, ten-item Connor–Davidson Resilience Scale; DASS-A, anxiety subscale of the 21-item Depression, Stress and Anxiety Scales (DASS-21); DASS-D, depression subscale of the DASS-21; DASS-S, stress subscale of the DASS-21; K6, six-item Kessler Psychological Distress Scale; LTE, List of Threatening Experiences; MCS, mental component scale of the 12-item Short Form Health Survey (SF-12); PCS, physical component scale of the SF-12; SLEs, personal stressful life events; TQ, Trypophobia Questionnaire.

### Univariate associations between trypophobia and severe psychiatric symptoms, functioning, distress and health-related quality of life

Compared with those without trypophobia, those with trypophobia were also significantly more likely to show severe symptoms of anxiety (19.1% *v.* 10.3%, OR = 2.06), depression (15.2% *v.* 8.4%, OR = 1.96) and stress (10.5% *v.* 5.7%, OR = 1.93); all *P* < 0.01. Trypophobia was also significantly associated with more days of lost productivity, higher levels of global distress and poorer mental health-related quality of life; all *P* < 0.01. There was no significant difference between the groups with respect to exposure to external stressors; *P* > 0.05.

### Multivariable logistic regression models showing the role of trypophobia in severe anxiety, depressive and stress symptoms

Findings from the separate multivariable analyses also showed that even after accounting for a wide range of personal and external factors, including resilience, SLEs, age, sex, personal and family psychiatric history, and adverse childhood experiences, trypophobia remained a significant factor in all three models (severe anxiety symptoms: OR = 1.83, 95% CI = 1.32–2.53; severe depressive symptoms: OR = 1.78, 95% CI = 1.24–2.56; severe stress symptoms: OR = 1.68, 95% CI = 1.11–2.53) (Table 2). Lower resilience, high stress exposure and childhood adverse experiences were also commonly associated with all three severe symptom outcomes; all *P* < 0.05. Personal psychiatric history was associated with severe anxiety and stress symptoms, and family psychiatric history was associated only with severe anxiety symptoms in the multivariable models (Table 2). Notably, whereas female sex was associated with higher odds of severe anxiety symptoms (OR = 1.44, 95% CI = 1.06–1.95), it was associated with lower odds of severe depressive symptoms (OR = 0.70, 95% CI = 0.50–0.96). The pseudo-*R*^2^ values for these models were 0.14 for severe depressive and anxiety symptoms and 0.09 for severe stress symptoms, respectively.

**Table 2:** Multivariable logistic regression models showing the contribution of high levels of trypophobia symptoms to severe anxiety, depressive and stress symptoms in the representative epidemiological youth sample

	Severe anxiety symptoms (DASS-A ≥15) (*n* = 246)			Severe depressive symptoms (DASS-D ≥21) (*n* = 199)			Severe stress symptoms (DASS-S ≥26) (*n* = 136)		
	OR	95% CI	*P*	OR	95% CI	*P*	OR	95% CI	*P*
Trypophobia									
No trypophobia (TQ < 31)	Ref.			Ref.			Ref.		
Has trypophobia (TQ ≥ 32)	**1**.**83**	**1.32**–**2.53**	**<0**.**001**	**1**.**78**	**1.24**–**2.56**	**0**.**002**	**1**.**68**	**1.11**–**2.53**	**0**.**014**
Intrinsic and extrinsic factors									
Resilience (CD-RISC-10)	**0**.**90**	**0.88**–**0.92**	**<0**.**001**	**0**.**85**	**0.83**–**0.88**	**<0**.**001**	**0**.**91**	**0.88**–**0.93**	**<0**.**001**
<2 SLEs	Ref.			Ref.			Ref.		
≥2 SLEs	**2**.**08**	**1.51**–**2.86**	**<0**.**001**	**1**.**46**	**1.02**–**2.09**	**0**.**040**	**1**.**76**	**1.18**–**2.62**	**0**.**005**
Background factors									
Age	0.96	0.91–1.01	0.15	1.00	0.94–1.06	0.97	1.05	0.98–1.12	**0**.**16**
Male	Ref.			Ref.			Ref.		
Female	**1**.**44**	**1.06**–**1.95**	**0**.**020**	**0**.**70**	**0.50**–**0.96**	**0**.**029**	1.00	0.69–1.47	0.99
No personal psychiatric history	Ref.			Ref.			Ref.		
Has personal psychiatric history	**1**.**96**	**1.30**–**2.94**	**0**.**001**	1.36	0.86–2.14	0.20	**2**.**23**	**1.39**–**3.57**	**0**.**001**
No family psychiatric history	Ref.			Ref.			Ref.		
Has family psychiatric history	**1**.**61**	**1.11**–**2.33**	**0**.**013**	1.02	0.66–1.59	0.92	1.25	0.78–2.00	0.36
Adverse childhood experiences	**1**.**32**	**1.16**–**1.50**	**<0**.**001**	**1**.**32**	**1.14**–**1.52**	**<0**.**001**	**1**.**36**	**1.17**–**1.59**	**<0**.**001**

Values significant at the *P* < 0.05 level are shown in bold.

CD-RISC-10, ten-item Connor–Davidson Resilience Scale; DASS-A, anxiety subscale of the 21-item Depression, Stress and Anxiety Scales (DASS-21); DASS-D, depression subscale of the DASS-21; DASS-S, stress subscale of the DASS-21; Ref., reference group; SLEs, personal stressful life events; TQ, Trypophobia Questionnaire.

### Effects of interactions between trypophobia and stress exposure on symptom severity

Having examined the influences of trypophobia on severe symptoms to detect youths at higher mental health risk, we further examined whether trypophobia would have an impact on the severity of the three symptom outcomes, and whether it would potentiate the impact of stress exposure on mental health. We therefore conducted two-way ANOVA for the presence of each of the three mental health conditions to examine the potential additive and interaction effects of trypophobia and stress exposure on symptoms of anxiety, depression and stress, adjusting for the same set of covariates as in the multivariable analyses above.

**Fig. 1:**
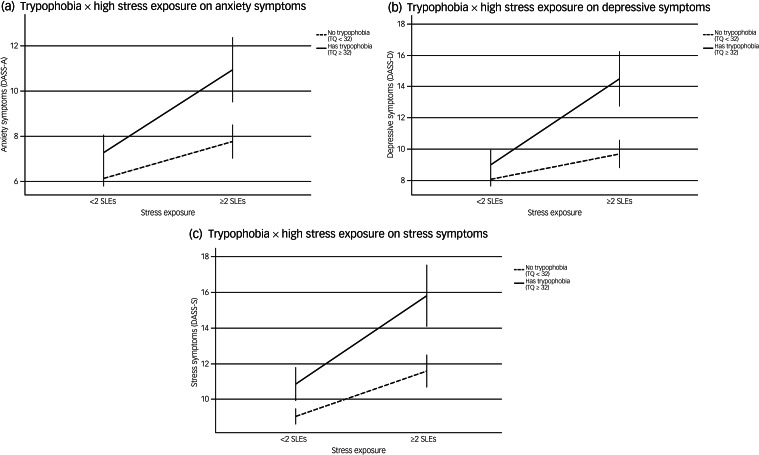
Interaction effects of trypophobia and stressful life events on the severity of anxiety, depressive and stress symptoms. Additive and interaction effects of trypophobia and high stress exposure on the severity of (a) anxiety symptoms, (b) depressive symptoms and (c) stress symptoms in the Hong Kong epidemiological youth sample. Age, sex, personal and family psychiatric history, childhood adversity and resilience were adjusted in all models. Main effects of trypophobia and high stress exposure were significant in all three models (*P* = 0.001). The trypophobia × stress exposure interaction effect was significant for depressive symptoms (*P* = 0.001) and marginally significant for anxiety (*P* = 0.053) and stress (*P* = 0.054) symptoms. DASS-A, anxiety subscale of the 21-item Depression, Stress and Anxiety Scales (DASS-21); DASS-D, depression subscale of the DASS-21; DASS-S, stress subscale of the DASS-21; SLEs, personal stressful life events; TQ, Trypophobia Questionnaire.

As well as significant main linear effects of trypophobia and high stress exposure (all *P* < 0.001), the effect of trypophobia × high stress exposure was also significant for depressive symptoms (*P* = 0.001) and marginally significant for anxiety symptoms (*P* = 0.053) and stress symptoms (*P* = 0.054). The additive and potentiating effects of trypophobia and stress exposure on the severity of the three symptom outcomes are illustrated in Fig. 1(a)–(c).

## Discussion

The phenomenon of trypophobia and its associations with mental health have not been extensively explored. To the best of our knowledge, this was the first study to examine the prevalence of trypophobia and its potential implications for mental health and functioning in a large-scale household-based epidemiological youth sample. We found that more than one in six young people (17.6%) in Hong Kong experience trypophobia, which closely resembles the rate reported in a previous sample of 405 community members in Bahrain (18.3%).^40^ In addition, we found that those with trypophobia were significantly more likely to have severe anxiety, depressive and stress symptoms, as well as poorer functioning and mental health-related quality of life. The associations between trypophobia and psychiatric symptoms remained even when accounting for both personal and environmental risk factors. Notably, although trypophobia was not associated with the frequency of SLEs experienced, we showed that it could significantly potentiate the effect of high stress exposure on mental health, particularly on depressive symptoms. This supports the perspective of trypophobia as reflective of enhanced neurobiological sensitivity to one's environment. The present study offers insights into the processes involved in the relationship between trypophobia and mental health, as well as the plausible clinical utility of trypophobia as a brief and less stigmatising marker of psychiatric risks for use with young people in future engagement, detection and intervention work.

Unlike other forms of phobia, the relationship of trypophobia with mental health and the underlying mechanisms remain unclear. The associations observed between trypophobia and visual stress in prior work have led some researchers to attribute its manifestation, at least partially, to the hyperexcitability of the visual cortex.^8^ It has been suggested that symptoms of trypophobia (e.g. discomfort) may initially be adaptive responses and form part of a homeostatic process that helps individuals by reducing the metabolic load on the visual cortex.^10,11^ The elevation in haemodynamic activities in posterior cortical areas in response to trypophobic stimuli among individuals with trypophobia has also been used to support such a hypothesis.^8^

Indeed, the associations between trypophobia and elevated mental health risks can be considered from the perspectives of visual processing and attention in relation to one's environment.^41,42^ Deficits in visual attention and disruptions in cortical networks have long been implicated in various psychiatric conditions, including symptoms on the psychosis spectrum.^43–45^ In a number of previous studies, the presentation of trypophobic stimuli has been found to elicit late positive potential amplitudes,^41,42^ which have been linked to poorer cognitive control during affective processing and excessive attention to emotional and unpleasant stimuli, as well as higher levels of internalising symptoms.^46^ From this perspective, it is plausible that individuals with trypophobia (characteristic of elevated cortical excitability) may be more likely to attend to and show greater reactivity to external stress, thereby further accentuating the impact on mental health. Notably, we found that although trypophobia and stress exposure were independently associated with all three symptom outcomes, the interaction effect of trypophobia and stress exposure was significant only for depressive symptoms and marginally significant for anxiety and stress symptoms, when accounting for a wide range of personal background factors. This suggests that there might exist some inherent differences in the nature of the three symptom dimensions. Indeed, a number of recent studies have found that depressive symptoms generally show greater reactivity to external stress compared with anxiety symptoms,^47^ and that the interaction effects of personal stressors and genetic risks may be more prominent for depressive symptoms.^48^ Further studies would be helpful to clarify the similarities and differences in the mechanisms underlying the three symptom dimensions, particularly in relation to the roles of cortical hyperexcitability (as reflected by trypophobia) and reactions to external stressors.

Our study had several important strengths. First, this was the first large-scale epidemiological study conducted to examine the prevalence of trypophobia and its effects on mental health and functioning. As well as offering insights into the phenomenon, our findings may contribute to the identification of a simple, low-stigma yet useful transdiagnostic marker of mental health risks with functional implications. As young people are often less likely to seek professional help for mental health needs,^15,46^ incorporating measures of trypophobia in future routine screening may improve both youth engagement and possible earlier detection of young people at greater psychiatric risk. Despite some overlaps with other phobias in symptom presentation and potential underlying neurobiological mechanisms, trypophobia is not considered to be a form of specific phobia in existing diagnostic systems.^17^ In support of this, various studies have reported that the discomfort induced by trypophobic stimuli is more consistently linked to disgust sensitivity than to fear.^9,10,49^ This has the benefit of limiting the stigma attached to trypophobia symptoms among community members. The present study also extended previous work suggesting specific phobias during childhood as early markers of future psychopathology^50,51^ to consider the role of trypophobia in other mental health outcomes. In addition, by accounting for a wide range of known risk factors, our multivariate models confirmed the significance of trypophobia in mental health. The study of its interactions with external stressors also offered preliminary evidence of the potential mechanisms underlying trypophobia, which could be explored further in future work.

To date, the study of intervention opportunities for trypophobia remains limited, although separate case reports have stated that cognitive–behavioural therapy and sertraline are effective in reducing physiological responses to trypophobic stimuli and in developing new coping behaviours in children and adolescents.^49–52^ It is hoped that our current findings could provide a basis for future studies to examine the phenomenon in greater depth. It remains to be explored whether the treatment of trypophobia symptoms could help reduce psychiatric risks and whether the treatment of anxiety and depressive symptoms could similarly reduce trypophobia symptoms.

We also acknowledge several limitations. Although this study provides some important information about general mental health risks and functional impairments associated with trypophobia, the long-term implications and potential predictive utility require further investigation. Longitudinal studies would be helpful to examine any possible bi-directional associations between trypophobia and psychiatric symptoms (e.g. whether they may be situational and time-limited or more reflective of a long-standing trait), as well as the possible course of trypophobia. A more in-depth study into the presentation of trypophobia symptoms, differentiating between fear and avoidance responses to trypophobic stimuli, as in other forms of phobia, may also offer further information about the severity of the condition and its implications for daily life functioning.

Despite being significant, the associations we observed between trypophobia and mental health outcomes were generally weak. Previous studies examining physiological markers such as heart rate variability have indeed found community studies to show weaker effects in relation to depression compared with clinical studies.^53^ Modest effects have also been reported in a previous epidemiological study in older adults for polygenic scores and SLEs as predictors of depressive symptoms (pseudo-*R*^2^ = 0.01 for both);^54^ these have nevertheless been taken as important results.

Indeed, the small effect sizes we found suggest that other factors may play more prominent parts in determining recent mental health outcomes in population-based samples. One of the reasons might relate to trypophobia being a ‘trait-like’ phenomenon (such that its symptoms might emerge at an early age),^11^ whereas measures of psychiatric symptoms used in our study (DASS-21) and in a number of prior studies^12,48^ (e.g. the seven-item Generalized Anxiety Disorder scale) capture past 1- or 2-week experiences of psychopathology, which may be subject to later life experiences. The presence of trypophobia may thus represent greater proneness to psychiatric risks, although whether one will present severe depressive or anxiety symptoms may depend on other factors, such as environmental stressors. Further studies on this topic in other population-based samples would be helpful to clarify the exact mechanisms underlying the relationship between trypophobia and mental disorders. It would be worthwhile to combine physiological measures with the Trypophobia Questionnaire to confirm findings in the existing literature. In addition, whereas female preponderance is generally observed for mood disorders, we found in this study that when trypophobia and other risk and protective factors were accounted for, being female was associated with severe anxiety symptoms, whereas being male was associated with severe depressive symptoms. Further analyses would help to elucidate the factors underlying the sex differences in relation to the two major symptom dimensions observed.

Young people today face unprecedented challenges to mental health. The early identification of those at risk for mental disorders using simple and less stigmatising markers can be critical to preventive intervention work in the population. Trypophobia may serve as a novel marker of not only increased mental health risks and functional impairments but also greater adverse responses to external stressors. Assessing trypophobia in future mental health screenings in the community and population may be considered, particularly in low-resource and time-limited settings.

## Supporting information

Wong et al. supplementary material Wong et al. supplementary material

## Data Availability

The data presented in the current manuscript and further information about the data can be made available upon reasonable request. Enquiries should be submitted to the corresponding author.
