# Supplementary material for: Excessive fear of clusters of holes, its interaction with stressful life events and the association with anxiety and depressive symptoms: large epidemiological study of young people in Hong Kong
Source: BJPsych Open. 2023 Aug 14;9(5):e151. doi: 10.1192/bjo.2023.540 (PMC10594086; doi:10.1192/bjo.2023.540)
Supplement: Wong et al. supplementary material [file S2056472423005409sup001.docx]

**Supplementary Material**

**Excessive fear of clusters of holes and its interaction with stressful life events on anxiety and depressive symptoms: a large epidemiological study of young people in Hong Kong**

Stephanie MY Wong^1#^, Eric YH Tang^1#^, Christy LM Hui^1*^, YN Suen^1^, Sherry KW Chan^1,2^, Edwin HM Lee^1^, KT Chan^1^, Michael TH Wong^1^, Arnold J Wilkins^3^, Eric YH Chen^1,2^*

^#^Equal contribution

^1^Department of Psychiatry, School of Clinical Medicine, LKS Faculty of Medicine, The University of Hong Kong, Hong Kong

^2^The State Key Laboratory of Brain and Cognitive Sciences, The University of Hong Kong, Hong Kong

^3^Department of Psychology, University of Essex, Essex, United Kingdom

***Corresponding authors**

**Prof Eric Y. H. Chen**

MA(Oxon), MBChB(Edin), MD(Edin), FRCPsych, FHKAM(Psychiatry)

(Chair Professor, Department of Psychiatry, The University of Hong Kong)

**Dr Christy L. M. Hui**

PhD(Psychiatry)

(Associate Professor, Department of Psychiatry, The University of Hong Kong)

Address: Department of Psychiatry, 2/F New Clinical Building, Queen Mary Hospital, Pokfulam Road, Hong Kong

Email: eyhchen.hk@gmail.com

**Supplementary Material A.**

##### Table S1. Differences in trypophobia symptoms and other sample characteristics between those without and with severe symptoms in the epidemiological youth sample (n = 2065)

|  |  | Anxiety symptoms | | |  | Depressive symptoms | | |  | Stress symptoms | | |
| --- | --- | --- | --- | --- | --- | --- | --- | --- | --- | --- | --- | --- |
|  |  | No-to-moderate  (DASS-A < 15)  (n = 1,819) | Severe  (DASS-A ≥ 15)  (n = 246) | *p* |  | No-to-moderate  (DASS-D < 21)  (n = 1,866) | Severe  (DASS-D ≥ 21)  (n = 199) | *p* |  | No-to-moderate  (DASS-S < 26)  (n = 1,929) | Severe  (DASS-S ≥ 26)  (n = 136) | *p* |
| Trypophobia | |  |  |  |  |  |  |  |  |  |  |  |
|  | Has trypophobia (TQ > 31), n (%) | **309 (17.0)** | **73 (29.7)** | **<0.001** |  | **324 (17.4)** | **58 (29.1)** | **<0.001** |  | **342 (17.7)** | **40 (29.4)** | **0.001** |
| Intrinsic and extrinsic factors | |  |  |  |  |  |  |  |  |  |  |  |
|  | Resilience (CD-RISC-10) | **24.49 (6.01)** | **19.59 (6.17)** | **<0.001** |  | **24.51 (5.90)** | **18.20 (6.44)** | **<0.001** |  | **24.22 (6.09)** | **19.45 (6.60)** | **<0.001** |
|  | ≥2 SLEs, n (%) | **323 (17.8)** | **86 (35.0)** | **<0.001** |  | **348 (18.6)** | **61 (30.7)** | **<0.001** |  | **361 (18.7)** | **48 (35.3)** | **<0.001** |
| Background factors | |  |  |  |  |  |  |  |  |  |  |  |
|  | Age | 19.77 (2.80) | 19.65 (2.84) | 0.50 |  | 19.76 (2.81) | 19.75 (2.72) | 0.96 |  | 19.73 (2.81) | 20.15 (2.68) | 0.094 |
|  | Female sex, n (%) | **1032 (56.7)** | **168 (68.3)** | **0.001** |  | 1088 (58.3) | 112 (56.3) | 0.58 |  | 1115 (57.8) | 85 (62.5) | 0.28 |
|  | Has personal psychiatric history, n (%) | **125 (6.9)** | **50 (20.3)** | **<0.001** |  | **138 (7.4)** | **37 (18.6)** | **<0.001** |  | **142 (7.4)** | **33 (24.3)** | **<0.001** |
|  | Has family psychiatric history, n (%) | **212 (11.7)** | **58 (23.6)** | **<0.001** |  | **233 (12.5)** | **37 (18.6)** | **0.015** |  | **239 (12.4)** | **31 (22.8)** | **0.001** |
|  | Has childhood adversity, n (%) | **580 (31.9)** | **131 (53.3)** | **<0.001** |  | **598 (32.0)** | **113 (56.8)** | **<0.001** |  | **634 (32.9)** | **77 (56.6)** | **<0.001** |

*Note.* Descriptive statistics are presented in the form of mean (SD), unless otherwise stated. Values significant at the *p* < 0.05 level are in boldface. CD-RISC-10 = 10-item Connor-Davidson Resilience Scale; DASS-A = anxiety subscale of the 21-item Depression, Stress and Anxiety Scales (DASS-21); DASS-D = depression subscale of the DASS-21; DASS-S = stress subscale of the DASS-21; SLEs = personal stressful life events; TQ = Trypophobia Questionnaire.
